# Trauma Exposure Across the Lifespan among Individuals Engaged in Treatment with Medication for Opioid Use Disorder: Differences by Gender, PTSD Status, and Chronic Pain

**DOI:** 10.21203/rs.3.rs-3750143/v1

**Published:** 2023-12-19

**Authors:** Monique N. Rodríguez, Dana D. Colgan, Sarah Leyde, Kenneth Pike, Joseph O. Merrill, Cynthia J. Price

**Affiliations:** University of New Mexico; Oregon Health & Science University; University of Washington Medical Center; University of Washington; University of Washington Medical Center; University of Washington

**Keywords:** Trauma, Post-Traumatic Stress Disorder, Chronic Pain, Gender Differences, Opioid Use Disorder, Medication Treatment

## Abstract

**Background:**

There is little study of lifetime trauma exposure among individuals engaged in medication treatment for opioid use disorder (MOUD). A multisite study provided the opportunity to examine the prevalence of lifetime trauma and differences by gender, PTSD status, and chronic pain.

**Methods:**

A cross-sectional study examined baseline data from participants (N = 303) enrolled in a randomized controlled trial of a mind-body intervention as an adjunct to MOUD. All participants were stabilized on MOUD. Measures included the Trauma Life Events Questionnaire (TLEQ), the Brief Pain Inventory (BPI), and the Posttraumatic Stress Disorder Checklist (PCL-5). Analyses involved descriptive statistics, independent sample t-tests, and linear and logistic regression.

**Results:**

Participants were self-identified as women (*n* = 157), men (**n** = 144), and non-binary (*n* = 2). Fifty-seven percent (*n* = 172) self-reported chronic pain, and 41% (n = 124) scored above the screening cut-off for PTSD. Women reported significantly more intimate partner violence (85%) vs 73%) and adult sexual assault (57% vs 13%), while men reported more physical assault (81% vs 61%) and witnessing trauma (66% vs 48%). Men and women experienced substantial childhood physical abuse, witnessed intimate partner violence as children, and reported an equivalent exposure to accidents as adults. The number of traumatic events predicted PTSD symptom severity and PTSD diagnostic status. Participants with chronic pain, compared to those without chronic pain, had significantly more traumatic events in childhood (85% vs 75%).

**Conclusions:**

The study found a high prevalence of lifetime trauma among people in MOUD. Results highlight the need for comprehensive assessment and mental health services to address trauma among those in MOUD treatment.

**Trial Registration:**

NCT04082637

## Introduction

A growing body of literature documents the high prevalence of trauma exposure and PTSD among individuals with substance use disorders (SUD) (1, 2), with rates of co-occurring post-traumatic stress disorder (PTSD) ranging from 33–50% (3). However, with the opioid overdose epidemic, more trauma-related research is needed as it relates to opioid use disorder (OUD), and relatively little is known about lifetime trauma exposure among persons engaged in treatment with medication for opioid use disorder (MOUD), critical for improving integrative and comprehensive care for this population (4).

An examination of traumatic events across the lifespan allows for differentiation between types of traumatic experiences (e.g., interpersonal, non-interpersonal, childhood), as well the opportunity to examine related sex/gender differences and the relationship to PTSD symptoms, all of which are crucial for understanding the impact of trauma on any health condition or population (5). For example, intimate interpersonal trauma is significantly more likely to be associated with symptoms of PTSD, when compared to non-interpersonal trauma and non-intimate interpersonal trauma (e.g., physical assaults perpetrated by non-intimates) (6), and there is a significant relationship between the number of traumatic events and the development of PTSD (7). Also, the potential health consequences of childhood trauma are increasingly evident. Systematic reviews consistently show a link between exposure to childhood violence and substance use disorder (8), with a 73% increased risk for SUD if there is a history of sexual abuse in childhood and a 74% increased risk if there is a history of physical abuse in childhood (9). Sexual trauma is more prevalent among women than men. Women with a history of sexual trauma are at increased risk for SUD compared to men (9) and have specific treatment-related needs due to the type of trauma endured and its impact on mental health (10).

In research specific to trauma for those with opioid use disorder (OUD) (N = 20,522), a recent systematic review examining child maltreatment demonstrated the high prevalence of childhood physical abuse in 43% in the total sample, and significantly more childhood sexual abuse among women (41%) compared to men (16%) (11). Studies specific to examining trauma among those treated with MOUD have been relatively limited in scope and/or small in sample size. For example, one study (N = 919) examined interpersonal trauma only (physical, sexual, or emotional abuse) and found that 23% reported sexual abuse, 43% physical abuse, and 58% emotional abuse and that there were no differences by gender on any of these categories (12). Unfortunately, this study by Powers did not distinguish whether the traumatic events occurred in childhood or as adults. Another study (N = 36) examined both interpersonal and non-interpersonal types of trauma (e.g., accidents, natural disasters) and found both to significantly predict OUD (13). A third study (N = 135) exami*n*ed current trauma only (over period of last 12 months) among those engaged in MOUD and found that more than one third reported interpersonal trauma (combining reported interpersonal traumas such as intimate partner violence, sexual assault, physical assault) and found similar overall rates among men (36%) and women (40%) (14).

Given the high prevalence of chronic pain among those in MOUD (15, 16), it is also highly relevant to examine the relationship between trauma exposure and chronic pain within this population. Prior research demonstrates that trauma exposure is associated with an increased risk of developing chronic pain (1, 2), defined as persistent pain lasting for at least three months that adversely affects the function or well-being of the individual (17). In addition, individuals with a trauma history are approximately three times more likely to develop a chronic pain condition than those without a trauma history (18). Within the population of individuals affected by chronic pain, individuals with a trauma history report more intense pain (19, 20), greater affective distress, and a higher disability (21, 22) than individuals without a trauma history. Previous research has also established a high comorbidity between PTSD and chronic pain in the general adult population (23), in veteran populations (24), and, most recently, among individuals engaged in MOUD (25, 26). Importantly, chronic pain has been identified as a significant contributing factor to SUDs, most notably OUD. Patients experiencing comorbid chronic pain and PTSD are reported to have increased odds of OUD compared to individuals with neither a chronic pain condition nor a PTSD diagnosis (26).

As noted above, the type of traumatic experience appears to matter; specifically, the type of traumatic experience appears to be differentially associated with the development of chronic pain. The relationship between exposure to non-interpersonal trauma (e.g., traumatic accidents) and the development of chronic pain is well-established in individuals with and without SUD, with research demonstrating that accident-related pain is associated with greater pain severity and related disability in those with vs. without SUD (27). The relationship between exposure to interpersonal trauma, childhood trauma in particular, and the development of chronic pain has also been established in the general population (28–30), replicated in SUD populations (31) and documented in OUD populations (32–36). However, in most studies examining chronic pain or OUD, childhood trauma exposure has been defined and limited to single types of childhood abuse or neglect (33, 37). Different types of trauma (e.g., interpersonal, non-interpersonal, adult and/or child, etc.) have yet to be investigated among persons with OUD. Doing so may illuminate important risk factors for those with co-occurring chronic pain and OUD (38).

The purpose of this study is to comprehensively examine lifetime trauma exposure among individuals engaged in treatment with MOUD. The four aims of this study are to: 1) examine prevalence of different types of trauma exposure among individuals in MOUD; 2) identify gender differences in lifetime trauma exposure; 3) examine whether trauma exposure and number of traumatic events predict PTSD diagnostic status and PTSD symptoms, and 4) compare types of trauma exposure among those with and without chronic pain.

## Methods

### Study Design and Enrollment

A NCCIH-funded randomized controlled trial to examine mindful body awareness training in individuals engaged in MOUD treatment provided the opportunity to examine the prevalence of self-reported lifetime trauma exposure and differences in trauma exposure by gender and among those with and without chronic pain. This study received Human Subjects Institutional Review Board approval from the University of Washington. Data for this project was collected at baseline, prior to randomization to study treatment groups. Study participants were recruited from five community clinics offering MOUD in Washington state.

Recruitment was based on referral of interested and potentially eligible patients by clinic staff (i.e., nurses, physicians, and counselors). The Research Coordinator at each clinical site screened for eligibility and enrolled patients interested in study participation. Screening criteria aimed to select patients with adequate treatment engagement and clinical stability to participate in the mindful body awareness intervention sessions. Evidence of medication dose stability: for buprenorphine/naloxone, this was defined as at least four weeks of medication treatment and an appointment frequency of less than once weekly. For methadone, this was defined as at least 90 days in treatment with a minimum dose of 60mg and no more than three missed doses or any missed dose evaluation appointments in the past 30 days. Patients also needed to speak English and be willing to attend intervention sessions when offered. They were excluded if they were unwilling or unable to remain in MOUD treatment for the one-year trial or if they showed evidence of overt psychosis or cognitive impairment.

### Measures

Demographic Characteristics, Health History and Substance Use History

Demographic characteristics, including self-identified gender, along with other information specific to health history, was collected by patient self-report. Substance use was assessed using the Timeline Follow-Back Interview (TLFB) (39); a calendar method used to identify substance use over the 90 days prior to study enrollment.

### Trauma History

The Trauma Life Events Questionnaire (TLEQ) was used to assess the prevalence and number of traumatic events across the lifespan (40). The TLEQ is a 23-item self-report measure to assess lifetime exposure to a broad range of potentially traumatic events (see appendix for items). Two sex-specific items were removed and not administered to participants: one specific to miscarriage and one specific to abortion. Participants are asked to report the number of times they experienced each event (event frequency) on a 7-point scale ranging from *never* to *more than 5 times*.

Based on the responses to the 21 item TLEQ, we chose to categorize the items as adult interpersonal trauma, adult non-interpersonal trauma, or childhood trauma, and then examined the type of event to determine if any could be combined conceptually to minimize the number of total categories for analysis (for example we combined natural disaster with other types of accidents to create a non-interpersonal category titled “accident”). We excluded 6 items from the original measure for which the response rate was relatively low; these were items 4 (military trauma), 6 (the survival of someone you loved after a life-threatening accident or illness), 7 (having had a life-threatening illness), 11 (witnessing a stranger beat, attack or kill someone), 19 (subjected to uninvited or unwanted sexual attention other than sexual contact covered by items 15, 16, 17, or 18), and 21 (experienced other events that were highly distressing such as lost in the wilderness; a serious animal bite; violent death of a pet; being kidnapped or held hostage; seeing a mutilated body or body parts). Our final set of 15 items and 11 categorizations are listed in Table 1.

### PTSD Symptom Severity

The Posttraumatic Stress Disorder Checklist for DSM 5 (PCL-5) assesses PTSD symptom severity (41). Participants were asked to indicate how much they have been bothered by each PTSD symptom in the past month. It includes 20 items with a 5-point scale ranging from 0 (not at all) to 4 (extremely). We used a screening cut-off of > 31, indicative of probable PTSD (42). The reliability of the PCL-5 in this sample was .93.

### Chronic Pain

The Brief Pain Inventory (BPI) (43) is a well-validated questionnaire comprising 11 items designed to evaluate the severity of pain and its impact on daily activities (i.e., pain interference) (44). The scale’s reliability in this sample was .88 for pain severity and .93 for pain interference.

### Analyses

Descriptive statistics (counts, percentages, mean values, and SDs) were used to summarize sample demographics, self-report indices, and survey scales. Independent sample t-tests were used to examine differences in trauma exposure between men and women and between those with and without chronic pain. Linear regression was used to examine whether the number of trauma events predicted PTSD symptoms. Logistic regression was used to examine whether the number of trauma events predicted PTSD status (scoring above the screening cut-point for PTSD). All analyses were conducted using Stata version 18.0 (College Station, TX, USA)

## Results

### Participants

This sample (N = 303) had a median age of 40, with ages ranging from 21–73. Self-report gender in the sample was 144 male, 157 female, and two non-binary. The majority (79%) of the sample identified as White, 9% as mixed-race, 5% as Black, 4% as Native American, 1% as Asian, and 1% as Native Hawaiian or Pacific Islander. Nine percent identified as Hispanic. The highest level of education was high school for 66% of the sample. Socioeconomic status was low, reflected in the overall low employment rate (34% employed (at either full or half-time) and high public insurance rate (72%) on Medicaid. Chronic pain was reported in 57% of the sample. Before study enrollment, most participants (67%) were engaged in MOUD treatment for over 12 months, reported high levels of abstinence from opioids and other substances, and had engaged in relatively few lifetime mental health services (see Table 2).

### Lifetime Trauma Exposure

All participants in the sample, with one exception, reported at least one lifetime traumatic event. Over 70% of the sample reported exposure to five types of traumatic events. Within the category of adult interpersonal trauma: 71% reported physical assault (e.g., robbed or witnessing a robbery when a weapon was used, or physically assaulted by a stranger), 79% reported intimate partner violence (IPV), and 89% reported the experience of a sudden and unexpected death of a close friend or loved one. Within the category of adult non-interpersonal trauma: 86% reported an accident (e.g., a natural disaster or injurious accident. Within the category of childhood trauma: 89% reported at least one type of traumatic event (see Table 3).

### Trauma Exposure and Gender

Women reported significantly more trauma than men in many categories (IPV, sexual assault, being stalked, total childhood violence, childhood witness of IPV, childhood sexual abuse, and sudden death of a loved one). Men reported significantly more trauma than women in witnessing a traumatic event, physical assault, and childhood physical abuse. Notably, despite gender differences the prevalence of exposure to some of these events was very high for both men and women; for example, IPV (men 73%; women 85%), physical assault (women 61%; men 81%), total childhood violence (men 72%; women 89%), and sudden death of a loved one (men 83%; women 94%). There was equivalent exposure to accidents across genders (see Table 3).

### Trauma Exposure and PTSD Status

In this study sample, 41% (n = 124) met the screening criteria for PTSD. Exposure to trauma was significantly higher across all categories of trauma for those positive for PTSD compared to those without, with the exception of childhood witnessing of IPV, accidents, or sudden death of a loved one (see Table 4). Notably, those with subthreshold symptoms of PTSD still reported exposure to a great deal of trauma; for example, 72% experienced IPV, 77% experienced childhood violence, and 64% reported physical assault.

### Number of Trauma Exposure Events and PTSD Symptoms and Status

**Figure 1:**
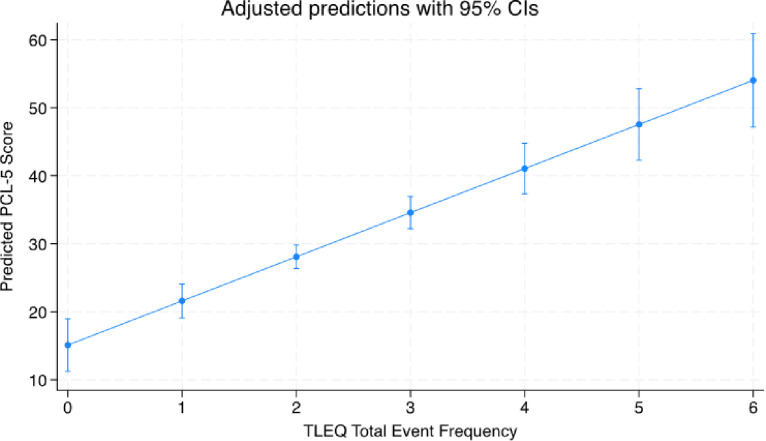
Legend not included with this version.

**Figure 2:**
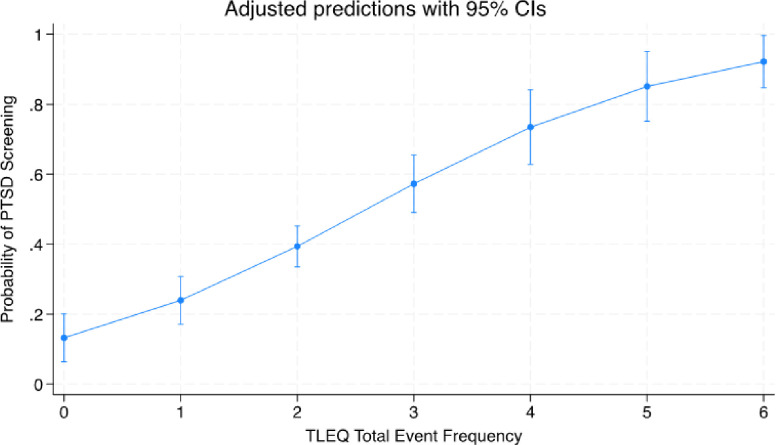
Legend not included with this version.

The number of reported traumatic events (i.e., the total number of events reported within each trauma category) predicted PTSD symptoms. Results from the univariate linear regression model showed that for every 1-point increase in number of trauma events, there was an increase of 6.5 on the PTSD symptom scale (β = 6.5, 95% CI, 4.8–8.2; Fig. 1). Likewise, the number of traumatic events predicted PTSD status (scoring above the PTSD screening cut-off on the PCL-5; OR = 2.1; 95% CI, 1.6–27.7; Fig. 2).

### Trauma Exposure and Chronic Pain

Individuals with chronic pain, compared to those without chronic pain, reported significantly more trauma in the following categories: accidents (n = 155, 91%), childhood violence (total; n = 147, 85%), childhood physical abuse (n = 82, 48%), witnessing IPV in childhood (n = 144, 78%), childhood sexual abuse (n = 106, 62%: Table 5).

## Discussion

This is the first study to examine lifetime trauma experiences among a large sample of individuals in MOUD. The results highlight the high prevalence of trauma in both childhood and in adulthood, as well as both interpersonal and non-interpersonal traumatic events in both men and women. While differences across gender and chronic pain status are notable, the remarkable prevalence of exposure to all trauma categories across all groups points to the critical need for both trauma assessment and mental health services that are accessible and integrated into MOUD treatment. Individuals in this sample were stabilized on MOUD for a substantial amount of time and reported high levels of abstinence from substance use yet were not accessing a level of mental health care commensurate with their need. Also notable is the particularly high report of sudden and unexpected death of a close friend or loved one – reflecting the tragic experience of loss among this sample likely due to drug overdose in their communities.

There were distinct gender differences in trauma exposure, the most striking being the higher number of women who reported sexual abuse in childhood and sexual assault in adulthood compared to men. This finding aligns with prior research and the identified need for women-specific programs in SUD treatment to address the high prevalence of sexual trauma (9, 10). Perhaps unexpected, although similar to study findings examining interpersonal trauma in the past 12 months among those in MOUD (14), was the high number of men who reported being victims of intimate partner violence (IPV); while not as high as the report of IPV among women, this finding warrants further research and clinical attention as it points to the need for more assessment and clinical support for IPV, for everyone regardless of gender/sex. Overall, these results point to the need to ensure that support services and trauma treatment are available and integrated into treatment to optimize outcomes for those receiving MOUD.

In this study, 41% of participants screened positive for PTSD, congruent with previously published literature (45, 46). Given the high prevalence of many types of traumatic experiences across the participants in this sample, we could not link PTSD diagnostic status to particular types of traumatic event (i.e., whether they occurred during childhood or as an adult; whether interpersonal or non-interpersonal). However, the results demonstrate the link between the number of traumatic events experienced and PTSD symptomatology and diagnosis. These findings align with previous studies (47), and the understanding that traumatic events in both childhood or adulthood can impact symptom severity, expression, and complexity (48).

The high prevalence of chronic pain in MOUD populations allowed us to examine the relationship between trauma exposure and chronic pain. Congruent with previous studies among individuals with and without SUD, our study found that individuals with OUD and chronic pain were more likely to report traumatic accidents (e.g., car accidents, falls, natural disasters) (27–30, 32, 33, 35–37, 49). Impaired cortisol secretion and psychological stress in response to a traumatic injury/ accident has been associated with development of chronic pain over time (27). Prior life circumstances that result in sustained, long term cortisol surges or activations, are known to contribute to cortisol dysfunction, and may then increase risk the risk of development of chronic pain (50). The relationship between abnormal physiological stress reactivity (i.e., heart rate, blood pressure, respiration rate, cortisol secretion) on negative health outcomes is well-established (51), and linked to pain somatization disorders (52, 53).

We also found that individuals who endorsed chronic pain were more likely to report childhood violence, including physical abuse, sexual abuse, and witnessing IPV in childhood. Most prior studies that have examined chronic pain, OUD, and childhood trauma exposure have been limited to single types of childhood abuse or neglect (33, 36). Our findings align with prior research showing a link between childhood trauma and chronic pain in community and SUD samples, highlighting the importance of assessing PTSD among those with chronic pain in MOUD and the potential need for psychological treatment in the context of recovery.

Providing trauma-focused therapy alongside treatment for opioid use disorder (46, 54), may prove to be beneficial. There is evidence that patients with chronic pain and a co-occurring history of physical trauma demonstrate a diminished response to treatment, when compared with a cohort of patients without a history of trauma. Moreover, recent clinical reports have described the indirect and successful treatment of intractable and chronic pain in patients with comorbid PTSD, only after instituting behavioral therapy targeting the PTSD symptoms. Cognitive-behavioral therapies with proven efficacy for the treatment of PTSD are now available to pain practitioners, and it is noteworthy that these interventions are now being tailored within comprehensive pain rehabilitation programs. Incorporating novel mindfulness and body therapy approaches to increase sensory and emotional awareness may also benefit individuals with PTSD and co-occurring OUD, and further research is needed in this area.

There are important related clinical implications of these findings for medical providers. Given the high prevalence of trauma exposure and PTSD among individuals with OUD, evidence-based PTSD screenings, assessments, and treatments should be provided alongside MOUD (55). Although calls to lower barriers and increase access to MOUD treatment have resulted in more primary care providers treating people with OUD (56–59) and national guidelines recommend that primary care clinics screen for depression (60) and anxiety (61), there is not a similar recommendation for universal PTSD screening (62) and, thus, detection rates are low (63, 64).

Study limitations include the characteristics of the sample: the majority were white, low SES, and from one region of the United States. The findings may not generalize to a more racially, ethnically or economically diverse population. Also, only two individuals in this study identified as non-binary, limiting our ability to learn more about this population and highlighting an important line of future research. The TLEQ, the questionnaire we used to collect trauma exposure data, is comprehensive and has been used in prior research; however, until there is a more standard measure used consistently across studies, it will continue to be challenging to compare findings from one study to another in order to gather a more subtle understanding of the sequelae of trauma exposure across the lifespan (5). This study has multiple strengths. First, it is a multi-site study including participants from urban and rural areas and multiple practice settings (opioid treatment program, mental health clinic, addiction clinic, and primary care clinic.) Patients reported a high proportion of days abstinent, and the majority had been in prolonged MOUD treatment, reducing the possibility that mental health symptoms were primarily substance-induced.

## Conclusions

In conclusion, the findings highlight the complex connection between trauma exposure, OUD, gender, PTSD symptoms, and chronic pain. This study provides valuable insights into the prevalence of trauma across genders and points to the potential impact on individuals engaged in MOUD. These findings may inform the development of enhanced gender-specific interventions and approaches for patients engaged in MOUD treatment, potentially addressing the interconnectedness of trauma, prolonged pain, and psychological issues in this population.

## Figures and Tables

**Table 1:** Trauma Categories and Corresponding TLEQ Items

Adult Interpersonal Trauma
Witness Trauma
10. Have you seen a stranger attack or beat up someone and seriously injure or kill them?
Intimate Partner Violence (IPV)
14. Have you ever been slapped, punched, kicked, or beaten up or physically hurt by your spouse or other intimate partner?
Physical Assault
8. Have you been robbed or present during a robbery where the robber used a weapon?
9. Have you ever been hit or beat up by a stranger or someone you didn’t know very well?
Adult Sexual Assault
18. Over 18 years old: did anyone touch sexual parts of your body or make you touch theirs without your consent?
Adult Stalking
20. Has anyone stalked you-in other words: followed you or kept track of your activities causing you to feel intimidated or concerned for your safety?
Sudden Death
5. Have you experienced the sudden unexpected death of a loved one?
Adult Non-Interpersonal Trauma
Accidents
1. Have you ever experienced a natural disaster?
2. Were you involved in a motor vehicle accident for which you received medical attention or that badly injured or killed someone?
3. Have you been involved in any other kind of accident where you or someone else was badly hurt?
Childhood Trauma
Childhood Physical Abuse
12. While growing up: Were you physically punished in a way that resulted in bruises, burns, cuts or broken bones?
Childhood Witnessing IPV
13. While growing up: Did you see or hear family violence?
Childhood Sexual Abuse
15. Before your 13th birthday: did anyone who was 5 years older than you, touch or fondle your body in a sexual way?
16. Before your 13th birthday: did anyone close to your age touch sexual parts of your body without your consent?
17. Between 13–18 yrs. old: did anyone touch sexual parts of your body or make you touch theirs without your consent?
Total Childhood Trauma
Items 12, 13, 15, 16, 17

**Table 2.** Sample Demographics

	Mean	Percent
Age, median (range)	40	(21–73)
Gender Identity		
Male	144	48%
Female	157	52%
Non-binary	2	1%
Hispanic	27	9%
Race		
Native American	13	4%
Asian	3	1%
Black or African American	16	5%
Hawaiian or Pacific Islander	4	1%
White	238	79%
More than one race	28	9%
Marital Status		
Married	33	11%
Single	215	71%
Domestic Partnership	18	6%
Unknown (Endorsed “Other”)	36	12%
Highest Education Level		
High school or less	168	44%
Two-year college/technical school	103	34%
College or advanced degree	32	11%
Monthly Income		
Less than $1000	179	59%
$1000 or more	124	41%
Employed	104	34%
Full time	69	66%
Part time	35	34%
Insurance^a^		
Medicaid	219	72%
Medicare	69	23%
Private	36	12%
None	5	2%
Chronic Pain 3 Months or More	172	57%
Mental Health Services in Lifetime		
0–10 therapy sessions	99	33%
11–30 therapy sessions	64	21%
>31 therapy sessions	140	46%
Time in MOUD Treatment Prior to Study Enrollment		
< 3 months	28	9%
3–6 months	26	9%
6–12 months	46	15%
> 12 months	203	67%
Percent days abstinent from any opioid		96%
Percent days abstinent from any substance**		88%
Medication for Opioid Use Disorder		
Methadone	35	12%
Buprenorphine	268	88%

a Respondents could select multiple responses.

** Percent Days Abstinent excludes cannabis, and prescribed buprenorphine or methadone

**Table 3.** Endorsed Trauma Categories

	N (%)	Female	Male	*P* value
N	301	157	144	
Witness Trauma	172 (57%)	75 (48%)	95 (66%)	0.001
Intimate Partner Violence (IPV)	238 (79%)	132 (85%)	104 (73%)	0.012
Physical Assault	214 (71%)	96 (61%)	116 (81%)	<0.001
Adult Sexual Assault	110 (37%)	90 (57%)	19 (13%)	<0.001
Stalking	161 (53%)	106 (68%)	53 (37%)	<0.001
Total Childhood Trauma	245 (81%)	140 (89%)	103 (72%)	<0.001
Childhood Physical Abuse	127 (42%)	61 (39%)	64 (44%)	0.373
Witnessing IPV	212 (70%)	117 (75%)	94 (66%)	0.079
Childhood Sexual Abuse	165 (54%)	115 (73%)	48 (33%)	<0.001
Accidents	260 (86%)	135 (86%)	123 (85%)	0.888
Sudden Death	269 (89%)	148 (94%)	120 (83%)	0.002

Binary variables, endorsed implies ≥ 1 on original 0–6 metric

Frequency (Percent%): p-value from chi-square test

**Table 4.** Endorsed Trauma Categories and PTSD Status

	Subthreshold Symptoms	PTSD	P value
N	179	124	
Witness Trauma	91 (51%)	81 (66%)	0.011
Intimate Partner Violence (IPV)	128 (72%)	110 (90%)	<0.001
Physical Assault	115 (64%)	99 (80%)	0.003
Adult Sexual Assault	55 (31%)	55 (45%)	0.011
Stalking	85 (48%)	76 (61%)	0.023
Total Childhood Trauma	137 (77%)	108 (87%)	0.022
Childhood Physical Abuse	64 (36%)	63 (51%)	0.011
Witnessing IPV	121 (68%)	91 (75%)	0.192
Childhood Sexual Abuse	83 (46%)	82 (66%)	<0.001
Accidents	151 (84%)	109 (88%)	0.384
Sudden Death	156 (87%)	113 (91%)	0.281

Binary variables, endorsed implies ≥ 1 on original 0–6 metric

Frequency (Percent%): p-value from chi-square test

**Table 5.** Trauma Categories Endorsed by Those With and Without Chronic Pain

	No Chronic Pain	Chronic Pain	P value
N	131	172	
Witness Trauma	70 (54%)	102 (60%)	0.314
Intimate Partner Violence (IPV)	101 (78%)	137 (80%)	0.609
Physical Assault	90 (69%)	124 (72%)	0.521
Adult Sexual Assault	43 (33%)	67 (39%)	0.276
Stalking	65 (50%)	96 (56%)	0.290
Total Childhood Trauma	98 (75%)	147 (85%)	0.020
Childhood Physical Abuse	45 (35%)	82 (48%)	0.020
Witnessing IPV	79 (61%)	133 (78%)	0.001
Childhood Sexual Abuse	59 (45%)	106 (62%)	0.004
Accidents	105 (80%)	155 (90%)	0.014
Sudden Death	113 (86%)	156 (91%)	0.225

Binary variables, endorsed implies ≥ 1 on original 0–6 metric

Frequency (Percent%): p-value from chi-square test

## List Of Abbreviations

SUD: Substance use disorder
PTSD: Post–traumatic stress disorder
OUD: Opioid use disorder
MOUD: Medication for opioid use disorder
TLFB: Timeline Follow–Back Interview
TLEQ: Trauma Life Events Questionnaire
PCL 5: Posttraumatic Stress Disorder Checklist for DSM 5
BPI: Brief Pain Inventory
IPV: Intimate partner violence
